# A unique nucleosome arrangement, maintained actively by chromatin remodelers facilitates transcription of yeast tRNA genes

**DOI:** 10.1186/1471-2164-14-402

**Published:** 2013-06-17

**Authors:** Yatendra Kumar, Purnima Bhargava

**Affiliations:** 1 Centre for Cellular and Molecular Biology, Council of Scientific and Industrial Research, Uppal Road, Hyderabad 500007, India

**Keywords:** Chromatin, Isw1, Isw2, Nucleosomes, RNA polymerase III, RSC, Transcription, Yeast tRNA

## Abstract

**Background:**

RNA polymerase (pol) III transcribes a unique class of genes with intra-genic promoters and high transcriptional activity. The major contributors to the pol III transcriptome, tRNAs genes are found scattered on all chromosomes of yeast. A prototype tDNA of <150 bp length, is generally considered nucleosome-free while some pol III-transcribed genes have been shown to have nucleosome-positioning properties.

**Results:**

Using high resolution ChIP-chip and ChIP-seq methods, we found several unique features associated with nucleosome profiles on all tRNA genes of budding yeast, not seen on nucleosome-dense counterparts in fission yeast and resting human CD4+ T cells. The nucleosome-free region (NFR) on all but three yeast tDNAs is found bordered by an upstream (US) nucleosome strongly positioned at −140 bp position and a downstream (DS) nucleosome at variable positions with respect to the gene terminator. Perturbation in this nucleosomal arrangement interferes with the tRNA production. Three different chromatin remodelers generate and maintain the NFR by targeting different gene regions. Isw1 localizes to the gene body and makes it nucleosome-depleted, Isw2 maintains periodicity in the upstream nucleosomal array, while RSC targets the downstream nucleosome. Direct communication of pol III with RSC serves as a stress-sensory mechanism for these genes. In its absence, the downstream nucleosome moves towards the gene terminator. Levels of tRNAs from different families are found to vary considerably as different pol III levels are seen even on isogenes within a family. Pol III levels show negative correlation with the nucleosome occupancies on different genes.

**Conclusions:**

Budding yeast tRNA genes maintain an open chromatin structure, which is not due to sequence-directed nucleosome positioning or high transcription activity of genes. Unlike 5′ NFR on pol II-transcribed genes, the tDNA NFR, which facilitates tDNA transcription, results from action of chromatin remodeler Isw1, aided by Isw2 and RSC. The RSC-regulated nucleosome dynamics at the 3′ gene-end serves as a novel regulatory mechanism for pol III transcription *in vivo*, probably by controlling terminator-dependent facilitated recycling of pol III. Salient features of yeast tDNA chromatin structure reported in this study can explain the basis of the novel non-transcriptional roles ascribed to tDNAs.

## Background

Positioning of the nucleosomes on specific locations with respect to factor binding sites can control the accessibility of underlying DNA and allow the transcription machinery to work effectively in a chromatin environment [1]. Genome-wide maps of nucleosomes in a number of eukaryotes have established that RNA polymerase II promoters are nucleosome- depleted but coding regions may have a regular array of statistically arranged nucleosomes [2-8]. The nucleosome-free region (NFR) just upstream of transcription start site (TSS), results from sequence preferences as well as actions of chromatin remodelers [9,10].

RNA polymerase (pol) III transcribes a heterogeneous set of small non-coding RNA genes with majority constituted of tRNA genes (70–130 bp). Earlier, active transcription of a tRNA gene was reported to exclude nucleosome from the gene *in vivo*[11]. Pol III-transcribed genes are found in comparatively nucleosome-depleted intergenic regions [4,6], which gives the general impression that chromatin may not have much to do with their transcription. Activation of pol III transcription from several sites after global nucleosome depletion in yeast cells, suggests otherwise [12]. Presence of chromatin remodelers RSC and Isw2 on pol III-transcribed genes has also been reported [13,14]. RSC shows different remodeling activity on pol II and pol III-transcribed genes [15] and regulates pol III-transcribed *SNR6* and *SUP4* genes [16,17]. Recent studies with human pol III-transcribed genes have reported that the presence of pol II and pol II-specific chromatin marks near pol III-transcribed genes generates an active chromatin milieu for them [18-20].

This study shows that nucleosome depletion mechanisms at NFR on tDNAs and 5’ NFR on pol II-transcribed genes are fundamentally different. A stronger upstream (US) nucleosome positioning, persistence of NFR under repression on most of the genes, sequence-independence of NFR and requirement of remodeler activities to maintain the chromatin structure in active state, are some of the observations which suggest tDNA NFRs are different from the 5’ NFR on pol II-transcribed genes. An array of Isw2-dependent, regularly spaced nucleosomes especially in gene upstream region reflects stronger US nucleosome barrier as compared to downstream of the gene terminator. We identify Isw1 as the new remodeler for pol III genes, which along with RSC maintains the tDNA NFR. Finally, pol III communication with RSC regulates the downstream nucleosome mobility. This downstream nucleosome dynamics can regulate the terminator-dependent pol III recycling and hence the rate of transcription.

## Results

### Chromatin structure of tDNAs in active and repressed states

**Figure 1:**
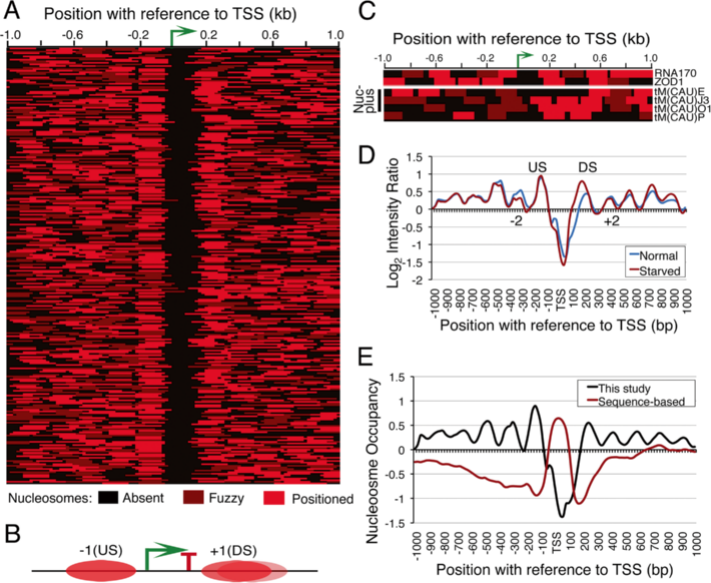
**Nucleosome arrangement around yeast tRNA genes. A**) Arrangements of nucleosomes around 273 yeast tRNA genes with respect to TSS (bent arrow) show NFR on gene regions. Cartoon **B**) gives the summary and typical arrangement of nucleosomes around a tDNA. **C**) Heat maps showing nucleosome arrangements around two non-tRNA genes *RNA170* and *ZOD1* and four initiator tRNA^Met^ (IMT) genes, three of which have fuzzy nucleosomes on the gene body (Nuc-plus). **D**) Average nucleosome profiles are compared between normal growth and starved condition for 52 genes that showed changes. **E**) In vivo nucleosome profile (this study) is compared with the sequence-based prediction of nucleosome profile [9].

We used high-resolution tiling microarray to map nucleosome positions around budding yeast tDNAs in a 2 kb window. A large number of positioned as well as fuzzy or delocalized nucleosomes (Figure 1A) were seen, which give similar profiles on alignment of the whole data with respect to TSS or TTS (transcription termination site) of all the tRNA genes, probably due to short transcribed length. Conforming to earlier reports [5,6], all tDNAs except three reside within a nucleosome-depleted region from −70 to +180 bp with respect to TSS (Figure 1B). However, some unique and interesting features associated with the gene-flanking nucleosomes can be noticed. Most tDNAs (82%) show a strongly positioned upstream (−1, US) nucleosome, centered at −140 bp position with respect to TSS while position of the downstream (+1, DS) nucleosome varies across the genes (Figure 1A, B; Additional file 1: Figure S1A). Nucleosomes in the further upstream regions are more regularly spaced compared to those in downstream of the genes. Similar NFR is seen on two other pol III-transcribed genes, *RNA170* and *ZOD1* (Figure 1C). We had earlier reported a similar arrangement on *SNR6* with NFR spanning over its TATA box and A box regions [17]. Such a common scheme of arrangement on rest of the non-tRNA genes or any special arrangement with reference to upstream TATA box or introns found in some of tDNAs is not seen.

Interestingly, nucleosomal arrangement is different on tRNA^Met^ family members. While EMT (elongator) genes show the general NFR-based arrangement of nucleosomes, three genes belonging to IMT (initiator) family show a fuzzy nucleosome covering the 5’ gene ends (Nuc-plus genes, Figure 1C, lower panel). This may be of functional significance, with a specialized role of chromatin in their regulation, as discussed later. We analyzed nucleosome positions on tDNAs in fission yeast and human CD4+ cells also from published datasets [2,7]. As depicted in Additional file 1: Figures S1B and C, tDNA chromatin in these organisms differs from the budding yeast. With a higher nucleosome density, ~50% of the genes in both species are embedded in nucleosomes and do not show clear NFR, strong US nucleosome or phased array (Additional file 1: Figure S1B,C). The tDNAs in human and mouse show tissue specific expression [20,21]. Non-uniform nucleosomal arrangement across all the genes could give differential expression of the genes in a tissue-specific manner.

Pol III transcription is repressed under stress conditions [22,23]. In order to see relevance of the tDNA chromatin structure to transcription, we used the same micro-array to find nucleosomal changes, if any near tDNAs, after prolonged nutrient deprivation. After 4 hr of starvation, nucleosomal changes are seen on 52 genes, within a window of 100 bp upstream and downstream of the gene body (Additional file 1: Figure S1D). An increase in average occupancy of both US and DS nucleosomes along with a small decrease in occupancy of −2 and +2 nucleosomes on both the sides can be noticed (Figure 1D). The average US nucleosome position is not disturbed while the gene terminator is encroached by the DS nucleosome, resulting in shrinkage of average NFR. This observation suggests that the DS nucleosome on the tDNAs either regulates their transcription or high transcription in active state keeps it away from the genes. However, NFR on rest of the genes is not disturbed under repression; suggesting nucleosome-depletion on active tDNAs is not a result of their high transcription rate.

### tDNA NFR is not sequence-directed

The 5’ NFR at pol II-transcribed genes is established with a significant contribution of sequence-preferences of nucleosome positions [9]. We asked whether NFR seen at tDNAs is directed because of sequence preferences of flanking nucleosomes. Sequence-based average nucleosome occupancy profile, as predicted (trained on real data) by Kaplan et al. around tDNAs [9], is opposite of the *in vivo* nucleosome profile found in this study (Figure 1E), indicating that nucleosome depletion on tDNAs is not because of underlying DNA sequences. Nucleosome density data from genome wide *in vitro* nucleosome assembly [24] also supports this observation and negates the possibility that variable positions of DS nucleosome are because of positioning signals (Additional file 1: Figure S1E,F). These observations suggest that nucleosome arrangement around tDNAs *in vivo* is maintained by active mechanisms, such as ATP-dependent chromatin remodeling, which help over-ride the sequence-directed nucleosome positioning information.

### Isw1 targets tDNAs and maintains tDNA chromatin in collaboration with Isw2

**Figure 2:**
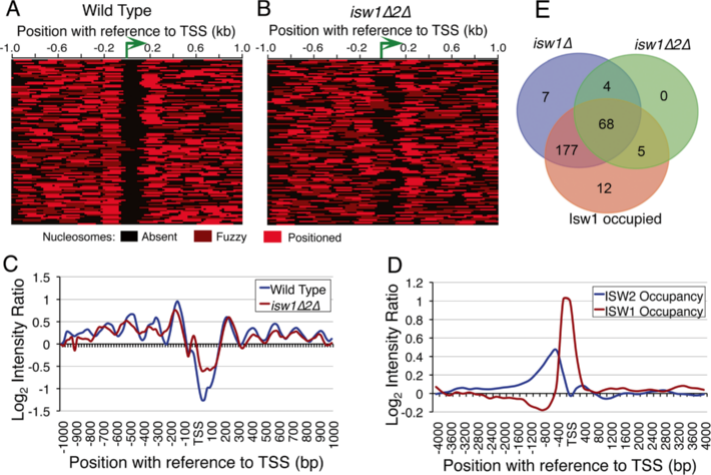
**ISWI maintains tDNA chromatin.** Heat maps in **A**) and **B**) compare the nucleosomes on 112 genes, which show nucleosome changes in *isw1∆2∆* cells compared to wild type cells. **C**) Comparison of average nucleosome occupancy profiles under two conditions as in panels **A** and **B**. **D**) Isw1 and Isw2 occupy different regions on yeast tDNAs [13,26]. **E**) Venn intersections among Isw1 target genes and the genes that show nucleosome gain on gene body either in *isw1∆* or *isw1∆2∆* cells.

Isw2 chromatin remodeler is recruited in upstream regions of tRNA genes by Bdp1 subunit of transcription factor IIIB [25]. We mapped nucleosomes in *isw1∆2∆* cells and used the published data [26,27] on remodelers as well as nucleosomes occupancy on tRNA genes in single deletion mutants (Figure 2; Additional file 2: Figure S2) to find their effect on these genes. A total of 112 genes show nucleosome gain (77 genes) or loss (35 genes) within 100 bp around the gene ends (cf. Figure 2A,B). Moreover, most of the genes show perturbation in upstream nucleosome array in *isw1∆2∆* cells (Figure 2B,C; Additional file 2: Figure S2A), probably because of Isw2 absence, since Isw2 mutation has been reported to disrupt chromatin structure in upstream regions of few tRNA genes [25] and in *isw2∆* cells, a disturbance in nucleosome array on tRNA genes is seen only in the upstream region (Additional file 2: Figure S2B). We found that majority of Isw2-occupied tDNAs are also enriched for Isw1 (Additional file 2: Figure S2C) but on different gene regions (Figure 2D). Only ~60% of 260 Isw2-occupied genes show nucleosome gain or loss in *isw1∆2∆* (Figure 2C; Additional file 2: Figure S2D). In contrast, most (249 out of 262) of the Isw1-occupied genes show nucleosome gain over gene body in isw1∆ cells, without disruption of flanking nucleosomal periodicity (Figure 2E; Additional file 2: Figure S2E). This observation indicates that Isw1 may be the major remodeler responsible for nucleosome-free status of tDNAs in wild type cells. It is intriguiging that when both Isw1 and Isw2 are deleted, the double deletion mutant shows nucleosome gain on far less number of genes (Figure 2E). This suggests that individual effects of deleting the remodelers are nullified on many genes probably due to the opposite directionality of remodelers’ actions. Isw2 aligns and pushes nucleosome from upstream towards TSS while Isw1 opposes its action by pushing the nucleosome away from the gene body. As a result of combined but antagonistic actions of Isw1 and Isw2, a nucleosome free region is created on the gene body along with strong positioning of the US nucleosome.

### RSC-pol III communication maintains the active state chromatin structure

**Figure 3:**
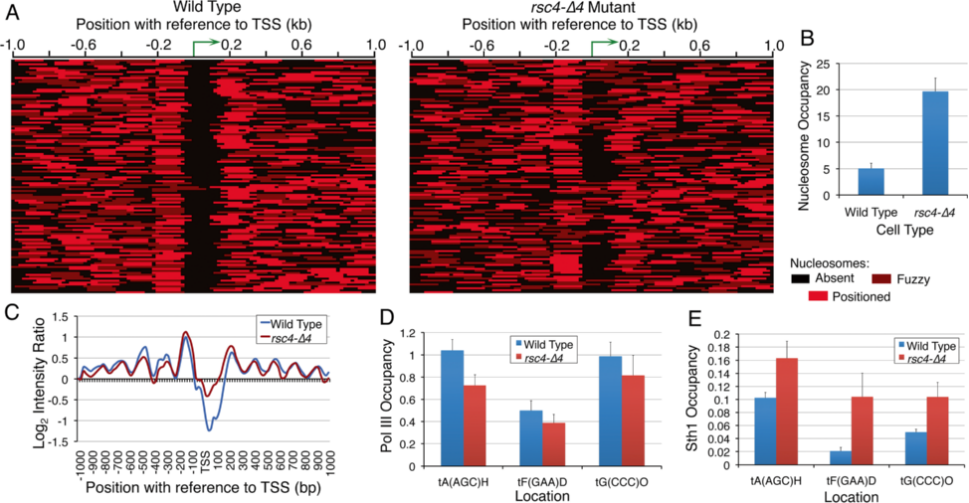
**NFR shrinkage and DS nucleosome dynamics in *rsc4-∆4 *mutant. A**) Nucleosome heat maps of 120 genes showing nucleosome changes in *rsc4-∆4* mutant as compared to the wild type cells. **B**) Real Time ChIP-PCR validation of a nucleosome encroaching the otherwise NFR, *tA(AGC)D* gene body. **C**) Comparison of the averaged nucleosome occupancy for 120 genes shown in the panel **A**. **D**) RPC160-myc (pol III) occupancy does not differ significantly in the two strains. **E**) RSC (Sth1-myc) occupancy shows somewhat higher levels in the mutant.

RSC is enriched on many tRNA genes [14] and compromising its catalytic activity was shown to cause increase in nucleosome density on these genes [15]. A mutation in the Rsc4 subunit of RSC is reported to abolish its interaction with pol III [28]. We had earlier shown that single nucleosomes near *SNR6* and *SUP4* are affected in localized manner in *rsc4-∆4* mutant [16,17]. Nucleosome mapping in this mutant revealed a large number of nucleosomal shifts (120 genes) on tDNAs (Figure 3A). Nucleosome in the mutant covers even the gene body in several cases (Figure 3A, right panel), as seen in the example of *tA(AGC)D*, validated by the ChIP-Real Time PCR measurement (Figure 3B). On the average, the US nucleosome position is not disturbed and most of the shifts are seen in the downstream region, where DS nucleosome encroaches the gene region while NFR shrinks (Figure 3C). Unlike *isw1∆2∆* mutant, periodicity in flanking regions is largely unaffected but nucleosomes show lower average occupancy on both sides of the NFR (Figures 3C). A similar gain in density of nucleosomes near pol III-transcribed genes upon loss of Sth1, the catalytic subunit of RSC was previously reported [15]. Pol III is not lost from the genes in this mutant as exemplified by three of the affected genes (Figure 3D). Similarly, the catalytic subunit Sth1 continues to occupy the genes in the *rsc4-∆4* mutant (Figure 3E), as reported even earlier [28]. This data indicates that the dynamics of the DS nucleosome seen in this mutant is guided by a cross-talk between RSC and pol III while bound to the genes.

### tDNA chromatin is required for optimum transcription

**Figure 4:**
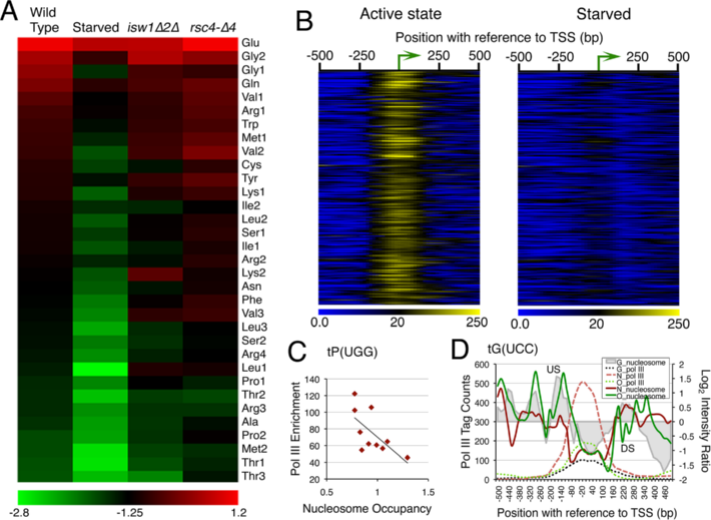
**Differential Pol III Occupancy and tRNA gene expression. A**) Heat map of tRNA levels showing perturbation of tRNA levels in wild type starved and remodeler mutants as compared to wild type active state levels. **B**) Heat maps show pol III tag counts binned around TSSs of all tDNAs in active and starved conditions. **C**) Scatter plot for nucleosome occupancy (−60 to TSS region) and pol III tag counts (−100 to +100 region) for isogenes of *tP(UGG)* family shows negative correlation. **D**) Pol III occupancy compared to gene-flanking nucleosome occupancies is shown for the three members of the tRNA^Gly^, *tG(UCC)* family. Genes denoted by G, N and O are color coded and US, DS nucleosomes are marked. Highest level of pol III on *tG(UCC)N* is complemented by the lowest nucleosome density and longest NFR.

We enquired whether nucleosomal perturbations seen in different conditions affect transcription by measuring cellular tRNA levels using Real Time qPCR analysis. Based on anti-codon sequence, budding yeast tRNA genes are grouped into 42 families, with highly similar sequences of multiple isogenes. Quantification of tRNA levels for 33 families in wild type cells (Additional file 3: Figure S3A; Additional file 4: Table S1) shows different intra-cellular tRNA levels across the families, which match with the codon usage of yeast cells [29], giving tRNA^Glu^ at the highest level, followed by tRNA^Gly^ (Additional file 3: Figure S3A). As compared to tRNA levels for these families (in a descending order) for wild type cells, the corresponding levels show gross perturbations under repressed or remodeler mutation conditions, like *isw1∆2∆* and *rsc4-∆4* cells (Figure 4A). Some families show higher levels in mutants probably because both gain and loss of nucleosomes on genes are observed in these mutants. Transcription of tDNAs is not affected due to Isw2 loss [13] probably because Isw2 on tDNAs maintains mainly upstream nucleosome periodicity (Figure 2). This indicates that the tRNA changes seen in *isw1∆2∆* cells are because of Isw1 absence, which targets the gene body. On the other hand, transcriptional changes in *rsc4-∆4* mutant could be due to changes in downstream nucleosome dynamics. This data shows nucleosome dynamics has profound influence on tRNA production.

### Pol III shows differential enrichment on isogenes

For the intra-cellular levels of tRNA species to be according to the copy number of the coding tRNA gene [29]; every gene copy within a family should be transcribed and occupied by pol III similarly. Different strengths of A and B-boxes may also result in different pol III levels on tDNAs. We used ChIP-seq method to measure the pol III level on different genes. Despite the sequence redundancy within and across the families, ChIP-seq data analysis with or without reallocation of non-unique reads [30] does not give significantly different results (Additional file 3: Figure S3B), probably because the average ChIP fragment size (~180 bp) is longer than the tRNA gene length in our experiment. No pol III on pseudo tRNA genes and high but differential pol III enrichment on all tRNA genes (Additional file 3: Figure S3C; Additional file 4: Table S2) is found. Pol III factors, TFIIIB and TFIIIC persist on genes after starvation [22] but a drastic loss of pol III is seen on all the genes (Figure 4B). Surprisingly, total pol III levels do not match with the relative RNA levels from the gene pools covered by each primer set (Additional file 3: Figure S3D). Moreover, different pol III levels on identical gene copies within a family (Additional file 3: Figure S3E,F) indicate different transcriptional activity on different isogenes. From these data, it appears that tRNA abundance (corresponding to copy number), is probably achieved by high transcription from few isogenes and not by equal transcription from all isogenes of a family.

### Nucleosome and pol III occupancy levels on tDNAs are inversely correlated

Pol III initiation factor TFIIIB binds at −30 bp from TSS and recruits pol III [31]. Differential pol III levels on isogenes could be because of different 5’ flanking sequences which affect TFIIIB binding [32-35]. Therefore, high nucleosome occupancy in this region would interfere with TFIIIB binding and as a result, pol III recruitment could be affected. Conversely, pol III presence on the genes could exclude nucleosomes. Hence, we compared nucleosome occupancy in upstream region (−60 to TSS) with pol III enrichment over gene region for tDNAs within each family. An inverse relationship (Pearson correlation between −0.5 to −0.9) for 16 families covering 135 genes (barring 1–2 outliers in each) was found. For example, the scatter plot for one of these families, tPro^UGG^ with 10 isogenes present on different chromosomes and Pearson correlation of −0.6544, shows greater enrichment of pol III near TSS of isogenes and lower nucleosome occupancy upstream (Figure 4C). In another example of the tGly^UCC^ family with only three members and Pearson correlation of −0.93, highest pol III levels are found on *tG(UCC)N*. The gene has lowest US but similar DS nucleosome occupancy when compared to *tG(UCC)G* and *tG(UCC)O*, which show comparatively lower pol III levels (Figure 4D). Thus, pol III occupancy (hence transcription) on most of the target genes has an inverse relationship with nucleosome occupancy in immediate upstream, which is not visible if average plots for individual familes are compared. It gets revealed better in studies of individual examples.

### DS nucleosome interferes with transcription from individual genes

**Figure 5:**
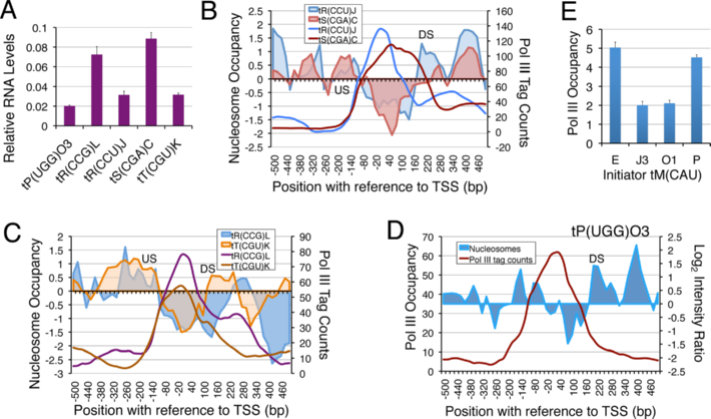
**DS nucleosome is inhibitory for transcription. A**) Relative RNA levels estimated by using five of the unique primer sets which could be designed for the five examples shown. Panels **B-D**) Comparison of nucleosome occupancy profiles with similar pol III levels on different genes. US and DS nucleosomes are marked. **B**) Comparison of nucleosome occupancy (shaded areas) profiles for *tR(CCU)J* and *tS(CGA)C* which have similar pol III levels (solid lines). Higher nucleosome density in downstream region and lower RNA levels of *tR(CCU)J* suggest that higher nucleosome occupancy in downstream region could be inhibitory for transcription. **C**) Comparison of nucleosome occupancy (shaded areas) profiles for *tR(CCG)L* and *tT(CGU)K* which have similar pol III levels. Higher nucleosome density in downstream region and lower RNA levels of *tT(CGU)K* suggest that higher nucleosome occupancy in downstream region could be inhibitory for transcription. The *tT(CGU)K* DNA has a shorter NFR and higher nucleosome occupancy even in the upstream region. **D**) On *tP(UGG)O3* gene, the high nucleosome occupancy on both the gene ends may be inhibitory for transcription, despite similar pol III levels. **E**) Pol III occupancy (fold enrichment over mock sample, from ChIP-seq data) on initiator tRNA^Met^ (IMT) genes is shown. Average with scatter from three independent experiments is plotted.

Nucleosome mapping results show that under different conditions, the US nucleosome is generally not disturbed. In comparison, the DS nucleosome shows greater dynamism (Figure 1D, 3C), suggesting it may have regulatory influence on gene transcription. Five more examples were studied, for which we could design unique primer sets for RNA estimation (Figure 5; Additional file 4: Table S1). Five of them are found on different chromosomes and belong to different families. RNA yield from *tP(UGG)O3* is lowest of the five (Figure 5A). Among the rest four, *tR(CCG)L* and *tS(CGA)C* RNA levels are higher than *tR(CCU)J* and *tT(CGU)K* RNA levels. The US nucleosome occupancy on all of them is found to be similar but pol III levels still show differences. The DS nucleosome occupancy on *tS(CGA)C* giving higher RNA yield, is lower than on *tR(CUU)J* with lower RNA yield (Figure 5B). Similarly, *tR(CCG)L* yields higher RNA and has lower DS nucleosome occupancy than *tT(CGU)K* (Figure 5C). In comparison to these, on *tP(UGG)O3*, pol III levels are comparatively low with both US and DS nucleosomes showing high occupancy (Figure 5D), which may be the reason for lowest RNA yield from this gene. A direct negative correlation of the DS nucleosome occupancy and pol III occupancy/RNA yield from other gene cases could not be made due to sequence redundancy problems associated with isogenes as mentioned above, but the differences in relative RNA levels from genes with mostly similar pol III occupancy can most probably be correlated with the DS nucleosome occupancy, as discussed later. It may be noticed in this context that on many genes, the DS nucleosome shows mobility towards the gene under repressed state (Figure 1C).

Different nucleosomal arrangement on initiator and elongator methionyl tRNA genes presents another interesting example of pol III gene regulation by nucleosome dynamics. Initiator tRNA^Met^ (IMT, Met1) with four identified isogenes is found at levels higher than the elongator counterpart (Additional file 3: Figure S3A; Met1 vs. Met2). Being initiator, high but regulated levels of this tRNA may be required by the cell for normal protein synthesis. It is interesting that out of four initiator tRNA (IMT) genes, those with a nucleosome covering the gene region show lower pol III enrichment compared to two other sequence identical copies (Figure 5E). Nucleosomes are generally repressive for transcription. Accordingly, lower pol III levels seen on the two initiator family members *(tM(CAU)J3* and *tM(CAU)O1)*, both of which have a fuzzy nucleosome covering their 5’ end and a very short NFR (Figure 1C); suggest either no or very low transcription from the two isogenes. The other two family members, *tM(CAU)E* and *tM(CAU)P*; which have higher pol III levels (Figure 5E) and more clear gene regions (Figure 1C) can probably meet the normal cellular requirements. At the same time, losing the nucleosome from the repressed copies provides a possibility of enhanced yield required under conditions of higher demand.

Above examples imply that the nucleosome occupancy and dynamics influence the pol III level and transcription from the tRNA gene loci. Different chromatin remodelers target different gene regions. Isw 2 maintains nucleosome arrangements in upstream region of tDNAs and Isw1 keeps the gene body nucleosome-depleted. RSC action is seen mostly in the downstream region, although the role of other remodelers can not be excluded at this stage. The DS nucleosome mobility towards the gene may inhibit the transcription by restricting the access of pol III to the gene terminator. Therefore, RSC activity keeps the DS nucleosome away from the TTS of the gene.

## Discussion

Sequence-directed nucleosome positions can be further modulated by DNA binding proteins and chromatin remodelers [36]. Nucleosome arrangement at tDNAs follows the same principles. Short pol III genes with intra-genic factor binding sites are mostly a size of single nucleosomal DNA. Transcription machinery of pol III has to compete with nucleosomes for access to the tRNA gene sequence, which favors nucleosome formation [9]. Protection of complete gene region by its transcription factors and high transcription rate have been implied as cause of nucleosome depletion at tDNAs. Persistence of tDNA NFR even after prolonged repression suggests that high transcription rate is not required for nucleosome depletion. Nucleosomes are rather actively excluded from the gene body by employing chromatin remodelers. The NFR at tDNAs is an elegant example where nucleosome-favoring sequences are depleted of nucleosomes *in vivo* by employing chromatin remodelers to facilitate transcription.

In yeast, several ATP-dependent chromatin remodelers are shown to direct the positions of most of the nucleosomes [26]. This study has shown the importance and novelty of the roles of two chromatin remodelers in tDNA regulation. Nevertheless, the nucleosome-depleted status of several gene bodies even in the ISWI double deletion mutant suggests the involvement of some other remodelers as well. We extracted and analysed the data on Chd1 and nucleosome occupancy on tDNAs from published studies [26]. Chd1 showed strong association with tDNAs but nucleosome profiles in wild type and *chd1∆* cells did not show discernible differences making the analysis results inconclusive. Isw2 activity in upstream region of tDNAs has been reported earlier [25,37], where it is found enriched [13]. Our study with double deletion mutant helped us find that Isw1 and Isw2 work in opposite manner on tDNA chromatin and it is the Isw1 action, which makes tDNAs nucleosome-depleted. The opposing actions of Isw1 and Isw2 position the US nucleosome strongly in most cases, which strongly supports the Isw2-dependent nucleosome periodicity in the further upstream region. The strong positioning of US nucleosome restricts the 3’ boundary of the upstream nucleosomal array to −70 bp, which could keep the −30 bp position nucleosome-depleted. This would facilitate TFIIIB binding and promote transcription in turn. RSC activity on the other hand, is seen in the gene downstream region. The earlier reported effects of RSC on tDNAs as judged by transcription of few genes, have been found in a condition where its catalytic subunit is lost [15]. We have used a RSC mutant in which RSC and its catalytic activity remain intact yet remodeling activity is lost due to its deficiency in interaction with pol III. Nucleosome positions and transcription output change across most of the genes in this mutant. This links RSC directly with pol III transcription and gives evidence in support of the mechanism by which RSC controls the tDNA activity and chromatin structure via modulating downstream nucleosome dynamics.

Regulation via nucleosome dynamics is apt for genes that are transcribed at very high rate since it can allow a quick regulatory response in adverse conditions and transcription can resume as soon as repressive condition is over. The interaction of pol III with RSC plays an important role in this by negotiating with the downstream nucleosome. Occluding the whole gene body and DS nucleosome dynamics offer novel regulatory mechanisms, adopted by a majority of pol III-transcribed genes in yeast. Transcription terminator facilitates the recycling of pol III on the same template giving faster re-initiation and hence transcription rate in vitro [38]. Blocking the gene terminator with a nucleosome can reduce the transcription by inhibiting the terminator-dependent recycling of pol III. In the absence of any known classical activation route available for these genes, facilitated re-initiation could be a very important mode of enhancing transcript yield in vivo. DS nucleosome mobility was shown to control the accessibility of TTS and transcription on the *SUP4* gene in vivo [16]. Thus, a differential dynamics of DS nucleosome on different tDNAs and their isogenes can lead to different transcript yields from and within a family, as observed in this study. Such a mechanism of gene regulation from downstream is not known for other classes of genes and could be a mechanism specially adapted by and evolved at pol III-transcribed loci. A DS nucleosome covering almost half of the gene body for most of tRNA genes in *S. pombe* may be significant in this connection (Additional file 1: Figure S1C).

The presence of a strong upstream nucleosome and NFR on tDNAs, which are found scattered across the genome may play a significant role in barrier functions attributed to tRNA genes [39] as nucleosome-free regions have been shown to serve as boundary elements [40]. Replication forks are reported to stall with high frequency near tRNA genes [41], probably due to the well-positioned US nucleosome on tDNAs seen in this study. The study enhances our perception about how nucleosome dynamics participates in pol III transcription regulation and how their arrangements on different gene classes have evolved to match the requirements.

## Conclusions

It is fully evident from this data that a strongly positioned upstream (US) nucleosome, the NFR on tDNAs and nucleosome downstream of the gene are not sequence-directed, rather the result of a fine balance between specific activities of the chromatin remodelers RSC and ISWI. Disturbance in this arrangement interferes with transcript yield but NFR on most of the genes is not lost under repression, suggesting the cause of nucleosome depletion is not the high transcriptional activity of tRNA genes. Collaboration between polymerase and ATP-dependent chromatin remodelers is important for optimum transcription from pol III-transcribed genes too. Involvement of other chromatin modifiers and histone modifications remains implicit. The strongly positioned US nucleosome and NFR on tDNAs can form the basis of the barrier functions reported for tRNA genes.

## Methods

### Strains and growth conditions

Details of yeast strains used in this study are given in Additional file 4: Table S3 [28,42-44]. Cells were grown to mid log phase (A_600_ ~0.8) in 100 ml 1X YEPD medium at 30°C. For setting up the repression, cells were peletted down in mid-log phase and shifted to pre- warmed 0.15X YEP medium without any carbon source and incubated for 4 hours [22].

### Tiling Array Design

A high density tiling array (244 K Chip) of Agilent platform containing 60-mer probes was designed. Probes covering all pol III-transcribed genes (70–120 bp length) with 1 kb upstream and 1 kb downstream gene regions were tiled at a resolution of 4 bases, giving a 56 bases overlap with the next probe. Non-unique probes that fell within the gene regions were not filtered out to prevent the possible loss of signal in all tRNA gene regions with highly similar sequences. As a corrective measure, the probes in gene and 200 bp immediate upstream or downstream regions were tiled with 59 base overlap instead of 56 bases, generating even higher resolution of 1 bp on and around the genes. This could allow us to identify the false nucleosomal signal, since any signal that covers only the gene region and not the flanks could be because of cross hybridization. A true nucleosomal signal would cover either upstream or downstream flank of the gene or both.

### Nucleosomal DNA preparation and array hybridization

Mono-nucleosomal and size-matched naked DNA samples were hybridized to the high resolution, custom-designed micro-array in triplicates. Mono-nucleosomal DNA was prepared by digesting formaldehyde-fixed chromatin with MNase according to previously described method [45] with minor changes. Briefly, spheroplasts were made from fixed cells in mid log phase, subjected to controlled MNase digestion and digested DNA was purified and checked on 1.25% agarose gels (Additional file 5: Figure S4A). To serve as a control, naked genomic DNA was digested with MNase such that it gave a fragment distribution ranging from 100–300 bp (Additional file 5: Figure S4B). The band corresponding to mono-nucleosomal DNA was cut out and purified from the gel. Equal amounts of mono-nucleosomal DNA and digested genomic DNA (2 μg) were labeled with Cy5 and Cy3 respectively, mixed and hybridized to array using manufacturer’s protocol. Post hybridization, slides were scanned with Agilent scanner and data extracted with Agilent Feature extraction software.

### Analysis of microarray data

Raw data from feature extraction files was imported into Agilent Chip Analytics software (Agilent Genomic Workbench Lite Edition 6.5). Signal intensities in both channels were normalized against the median of intensities at blank spots followed by intra-array intensity dependent Lowess normalization. Biological replicates showed good reproducibility as indicated by Pearson correlation and box-plots for log2 intensity ratios (Additional file 5: Figure S4C). Log_2_ intensity ratios from replicates were averaged for each probe and imported to MATLAB for peak calling. A previously used hidden Markov model [45] was applied for detection of nucleosomes through MLM package [46] in MATLAB, which suited well for our experiment and design as it is faster and allows nucleosome detection from incontiguous regions tiled in our array. Signals were smoothed by 3 point smoothing window and nucleosome peaks were called in different datasets. Nucleosomal peaks were then binned around TSS and terminator of the genes in question in 20 bp bins. Matrix files resulting from binning were used to make heat maps and clustering in Multiple Experiment Viewer [47]. Out of 275 tRNA genes, probes in microarray for two of them, *tK(CUU)C* and *tM(CAU)C* failed. These genes are located close together and we found aberrant hybridization signals from this region in a previous genome wide study too [5], suggesting the DNA sequence in this region may be problematic (data not shown). The bin-wise averages of log_2_ intensity ratios (nucleosomal DNA/genomic DNA) in the given window from microarray data were plotted against the distance from the reference point (TSS or TTS).

### Validation of Nucleosome shifts

Nucleosomal and ChIP DNA from independent preparations was quantified by Real Time quantitative PCR (ABI Prism) for validation of nucleosome shifts in different conditions. Genomic DNA sample served as a common calibrator. Primers were designed for selected genes (Additional file 4: Table S4) and all samples were diluted to same concentration (5 ng/ml). Data was normalized to a region in telomere at the right arm of chromosome 6 (TELVIR), as this region does not show any significant change in nucleosome occupancy under the conditions in question.

### Pol III ChIP and deep sequencing

Samples were prepared from 100 ml cultures of RPC128-FLAG tagged strain grown to A600 0.8-0.9 for pol III ChIP as described earlier [16]. Sepharose CL4B beads served as no-antibody control (mock) while, Anti-FLAG M2-Agarose was used to immuno-precipitate the DNA cross linked to FLAG tagged RPC128. Quality of ChIP samples prepared was checked with quantitative real time PCR on known target genes. Libraries for single end sequencing were made according to standard protocol from Illumina for mock and IP samples. Two biological replicates for IP and one for mock sample in each condition were sequenced using Illumina GA-II platform.

### Pol III ChIP-Seq data analysis

Raw data on 36 bp reads from single end sequencing were preprocessed with FASTX-Toolkit [48]. Reads having a quality score of at least 20, on at least 80% of the bases, were exported for further analysis. Reads were aligned to the yeast genome (sacCer3 assembly) with BOWTIE [49], allowing only unique and best alignments for each read. Reads were extended to average ChIP-fragment size (~180) and coverage on each base in the genome was calculated. Read coverage across the genome was scaled to one million and normalized to the total number of reads in each dataset so that comparisons between the samples can be done. Pol III peaks were called in HOMER package [50]. Reproducibility of replicates was checked with USeq package [51] using a scanning window of 500 bp with a step size of 250 bp and a minimum window score of 5.

Considering the very high degree of sequence similarity among tRNA genes, we aligned the reads to yeast genome with BWA [52] allowing 3 mismatches and multiple alignments for non-unique reads (multi-reads), up to 20 places in the genome. Next, multi-reads were reallocated to the most probable target positions according to a previously described algorithm [30] followed by calculation of coverage across the genome and peak calling by HOMER. This algorithm assigns non-unique reads to one of the candidate mapped locations, based on which location gets higher number of uniquely mapped reads. Comparing results from two strategies of alignment, we did not find any significant difference in the results which is attributable to a very small size of pol III target genes in comparison to the typical ChIP fragment size used.

### Nucleosome maps in fission yeast and human CD4 cells

Raw sequence reads from MNase-seq experiments [2,7] were quality filtered as described above. Filtered reads were aligned to respective genomes allowing 2 mismatches and only uniquely aligning reads were considered. Aligned reads were extended to 150 bp and read coverage for each base in the genome was calculated. Nucleosomal peaks were called with GeneTrack [53] (sigma: 20; exclusion: 147). Heat maps in Additional file 1: Figure S1B and C show nucleosomal peaks binned around tRNA genes. Sequences and annotations used for *S. pombe* were downloaded from PomBase [54] as on 01/09/2012. For human dataset, “hg18” assembly from UCSC was used.

### Nucleosome changes in Isw1 and Isw2 mutants

For comparison of nucleosome density in wild type and isw1∆ cells we used Raw MNAse-seq reads from published data [27]. The dataset had reads from *S. cerevisiae and S. paradoxus.* We aligned the sequencing reads competitively to both species by indexing both the genomes together using Bowtie. We made sure that a read was aligned only to one of the two genomes. Alignments to budding yeast were taken and genome wide coverage was calculated for each base as described above. Data for *isw2∆* cells was obtained from the authors’ website [37] and bin-wise averages of normalized log_2_ intensity ratios (nucleosomal DNA/genomic DNA) for tRNA genes were plotted.

### Analysis of Isw1 ChIP-chip data

2-color ChIP-chip data for Isw1 ChIP was taken from a previous study [26]. Data from three replicates were quantile normalized and log transformed using biotoolbox [55]. Normalized log_2_ ratios were binned around the tRNA genes TSS in appropriately sized bins and data matrix used for generating heat maps or average intensity plots.

### Quantification of tRNAs by real time PCR

Total RNA was extracted from 100 ml mid log phase cultures by acidic hot phenol method followed by DNase I treatment. RNA was then subjected to poly-A tailing with poly-A polymerase (Epicenter) using manufacturer’s protocol. First strand cDNA synthesis was performed by AMVRT primed with oligo-dT (18-mer) oligo followed by degradation of template RNA with RNAse A. The cDNA was extracted with Phenol:Chloroform:Isoamyl- alcohol, ethanol precipitated and quantified by Real Time qPCR using ∆∆Ct method. An equivalent amount of poly-A tailed RNA was kept as a control of reverse transcription reaction to make sure DNA contamination did not affect the quantification. U4 snRNA, a pol II transcript was used as internal control [17]. A common genomic DNA sample (5 ng/mL) was used as calibrator so that tRNA expression values could be compared between the gene families, without a complication due to copy number variation. Close to 100 specific primers for tRNA genes were designed with PRISE [56] covering all 42 families and tested for specificity and efficiency in real-time q-PCR using serial dilutions of cDNA templates. Finally 33 primer sets could be validated for use (Additional file 4: Table S1). Most of the primer sets are not unique to isogenes, and measure the transcript and mature tRNA levels together.

## Competing interests

The authors declare that they have no competing financial interests.

## Authors’ contributions

PB conceived the study and wrote the manuscript, PB and YK designed the experiments; YK performed the experiments and analyzed data. Both authors read and approved the final manuscript.

## Supplementary Material

Additional file 1: Figure S1 A prominent NFR at gene region is a characteristic feature of yeast tRNA genes. **A**) Heat maps show nucleosomes aligned according to TSS of 270 tDNAs, plotted by arranging the genes according to first, the DS nucleosome distance from TTS and then according to the families. One nucleosome can be always found in a narrow window upstream of the TSS (position marked by a bent arrow) whereas downstream nucleosome does not align to the same position according to TSS or TTS. Variations if any, in positions of US/DS nucleosomes and lengths of NFRs at gene region are better visible in this panel. A small number of genes, with DS nucleosomes farthest from TTS, are marked as “Select” by a short bar on right hand side of the panel. **B**) Heat maps for nucleosomes on 538 out of total 631 tRNA genes in human resting CD4+ cells were extracted from Schones et al. [7] and occupancy scores are given as color gradients. **C**) Heat maps for nucleosomes on 125 out of total 170 tRNA genes in *S. pombe* were extracted from Givens et al. [2]. D) Heat map of nucleosome profile after 4 hrs of nutrient starvation. Genes are arranged similar to panel A. Nucleosome dynamics mainly at 3’ end of several genes becomes evident on comparison of the two panels. E) and F) Bin-wise log_2_ intensity ratios for nucleosome signals on both sides for the tRNA genes according to TSS or TTS are plotted. The data was extracted and analyzed from the Zhang et al. [24]. Averaged data for all the 270 genes or a set of 30 genes (Select, panel A) are plotted together. The plots in both the panels show the presence of a nucleosome on the gene bodies of all 270 genes. An additional downstream nucleosome seen on the select genes suggests the variable position of the DS nucleosome is also not sequence-directed. Click here for file

Additional file 2: Figure S2 Nucleosome profile over tRNA genes in *isw1∆2∆* mutant. **A**) Normalized log_2_ [nucleosomal DNA/genomic DNA] intensity ratios for nucleosomes were binned around the TSS and TTS and averaged for all the 270 genes in wild type or *isw1∆2∆* cells. Perturbation of the upstream nucleosomal array is evident in case of the mutant. **B**) Figure shows normalized log_2_ ratios [nucleosomal DNA/genomic DNA] binned around the TSS of tRNA genes in wild type and *isw2∆* strain from Whitehouse et al. [37]. In this dataset, a sharp signal over tRNA gene body is also seen, which most likely is an artifact of array design as it is also obsereved in another dataset by Lee et al. [5] which used the same microarray. **C**) Venn intersections of the genes occupied by Isw1 or Isw2 [13,26]. **D**) Venn intersection of genes occupied by Isw2p and 112 genes showing nucleosome changes near them in *isw1∆2∆* mutant cells. **E**) Comparison of nucleosome profiles on 273 tDNAs in wild type and *isw1∆* cells according to available data [27] shows NFR on tDNA is occupied by a nucleosome in the mutant, which could be same as sequence-directed nucleosome on tDNAs (Figure 1E, Additional file 1: Figure S1E-F) [9,24]. Click here for file

Additional file 3: Figure S3 Pol III and nucleosome occupancy influence transcription. **A**) RNA levels normalized for copy number of genes by using genomic DNA as calibrator for Real Time PCR quantification of the cDNA for 33 tDNA families, relative to U4 snRNA. Family name descriptions and primer sequences are given in the Additional file 4: Table S3. **B**) Data analysis using either unique reads only or multi-reads reallocated to the loci, gives similar results. Normalized tag counts from pol III ChIP-seq experiment were calculated in two ways. All unique reads represent tag coverage binned around TSS and averaged for all genes, using only uniquely aligned reads. All reallocated reads represent tag coverage taking unique reads along with non-unique reads that map up to 20 places in genome and reallocating the non- unique reads probabilistically using a previously reported algorithm [30]. Both the plots show high overlap as tRNA genes although repetitive in sequence, are very small in comparison to the ChIP fragment size used in typical ChIP experiments. **C**) Normalized and averaged tag coverage profiles of all tDNAs and pseudo-tRNA (Additional file 4: Table S2) for RPC128-FLAG IP and mock samples. **D**) Family wise total pol III tag counts for all the genes that are covered in panel A and Figure 4A. Pol III tag count in a region −100 to +100 bp of every gene was averaged. Then for each primer set, this average pol III count is summed up for all genes that are covered by the primer set. **E**) Pol III fold-enrichment over mock from ChIP-Seq data for selected genes showing very high to very low pol III levels. **F**) The data in panel E is validated by ChIP-Real time PCR quantification for pol III on the same genes. Pol III occupancy data for both IP and mock samples are compared with the same on TELVIR region. Click here for file

Additional file 4 **Tables listing strains and primers used in this study.Table S1.** List of all primers and their sequences, used for tRNA expression analysis by Real Time PCR quantification method. Primers unique for five tRNAs are highlighted. **Table S2.** Details of tDNA like elements (pseudo-tDNAs) in yeast genome. Data is extracted from “pol3- scan” data [57]. **Table S3.** Details of yeast strains used in this study. **Table S4.** Details of primers used for quantifications in ChIP assays. Click here for file

Additional file 5: Figure S4 Quality control of microarray samples and data. (**A**) Quality check for the mono-nucleosomal DNA preparation. Lane M shows 100 bp DNA ladder used as molecular size marker. Purity of DNA before (lane 1) and after (lane 2) MNase digestion is shown. **B**) Genomic DNA control for nucleosome mappings after MNase digestion was checked. M shows the 100 bp DNA ladder used as size marker. **C**) Box plots showing near identical distribution of normalized Log2 ratios among three biological replicates. Pearson correlations between all possible replicate-pairs were 0.97 to 0.99, which confirmed the reproducibility of biological replicates. Click here for file

## Accession codes

All datasets from this study have been submitted to NCBI Geo for public access. Microarray data is linked to accession ID-GSE39802 and pol III ChIP-seq data to accession ID-GSE39566.
