# Supplementary figures and images for: A unique nucleosome arrangement, maintained actively by chromatin remodelers facilitates transcription of yeast tRNA genes
Source: BMC Genomics. 2013 Jun 17;14:402. doi: 10.1186/1471-2164-14-402 (PMC3698015; doi:10.1186/1471-2164-14-402)

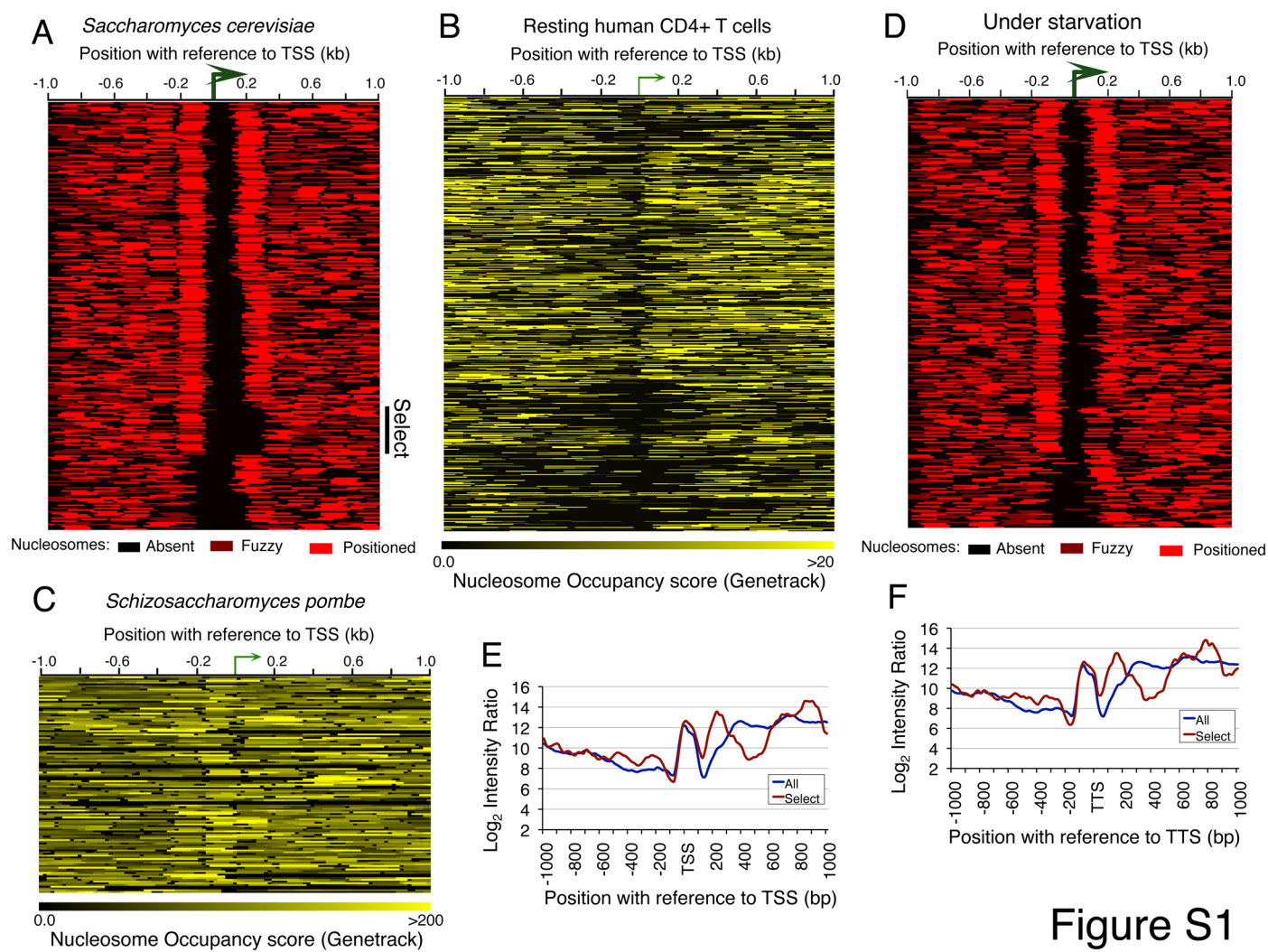

Figure S1

Supplement: Additional file 1: Figure S1 — A prominent NFR at gene region is a characteristic feature of yeast tRNA genes. A) Heat maps show nucleosomes aligned according to TSS of 270 tDNAs, plotted by arranging the genes according to first, the DS nucleosome distance from TTS and then according to the families. One nucleosome can be always found in a narrow window upstream of the TSS (position marked by a bent arrow) whereas downstream nucleosome does not align to the same position according to TSS or TTS. Variations if any, in positions of US/DS nucleosomes and lengths of NFRs at gene region are better visible in this panel. A small number of genes, with DS nucleosomes farthest from TTS, are marked as “Select” by a short bar on right hand side of the panel. B) Heat maps for nucleosomes on 538 out of total 631 tRNA genes in human resting CD4+ cells were extracted from Schones et al. [7] and occupancy scores are given as color gradients. C) Heat maps for nucleosomes on 125 out of total 170 tRNA genes in S. pombe were extracted from Givens et al. [2]. D) Heat map of nucleosome profile after 4 hrs of nutrient starvation. Genes are arranged similar to panel A. Nucleosome dynamics mainly at 3’ end of several genes becomes evident on comparison of the two panels. E) and F) Bin-wise log2 intensity ratios for nucleosome signals on both sides for the tRNA genes according to TSS or TTS are plotted. The data was extracted and analyzed from the Zhang et al. [24]. Averaged data for all the 270 genes or a set of 30 genes (Select, panel A) are plotted together. The plots in both the panels show the presence of a nucleosome on the gene bodies of all 270 genes. An additional downstream nucleosome seen on the select genes suggests the variable position of the DS nucleosome is also not sequence-directed. [file 1471-2164-14-402-S1.pdf]

Figure S2

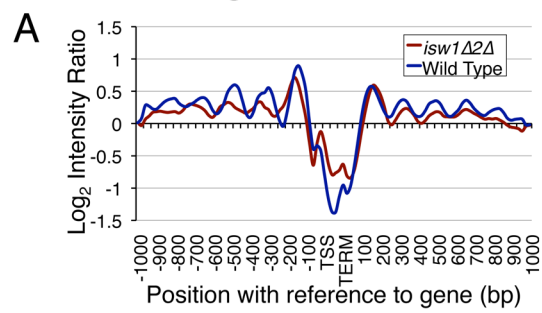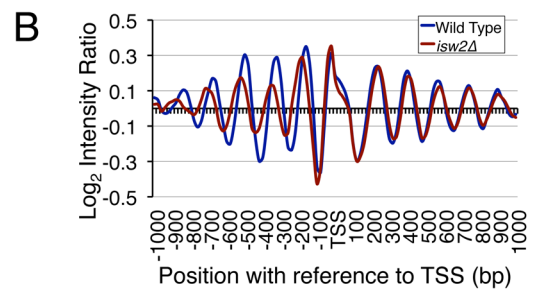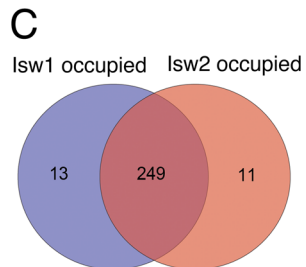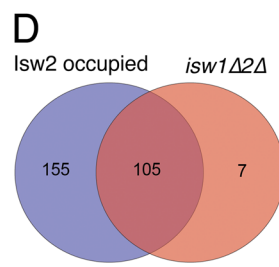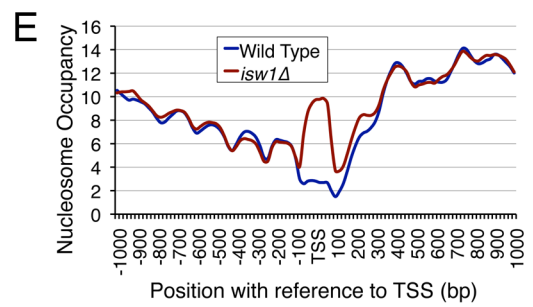

Supplement: Additional file 2: Figure S2 — Nucleosome profile over tRNA genes in isw1∆2∆ mutant. A) Normalized log2 [nucleosomal DNA/genomic DNA] intensity ratios for nucleosomes were binned around the TSS and TTS and averaged for all the 270 genes in wild type or isw1∆2∆ cells. Perturbation of the upstream nucleosomal array is evident in case of the mutant. B) Figure shows normalized log2 ratios [nucleosomal DNA/genomic DNA] binned around the TSS of tRNA genes in wild type and isw2∆ strain from Whitehouse et al. [37]. In this dataset, a sharp signal over tRNA gene body is also seen, which most likely is an artifact of array design as it is also obsereved in another dataset by Lee et al. [5] which used the same microarray. C) Venn intersections of the genes occupied by Isw1 or Isw2 [13,26]. D) Venn intersection of genes occupied by Isw2p and 112 genes showing nucleosome changes near them in isw1∆2∆ mutant cells. E) Comparison of nucleosome profiles on 273 tDNAs in wild type and isw1∆ cells according to available data [27] shows NFR on tDNA is occupied by a nucleosome in the mutant, which could be same as sequence-directed nucleosome on tDNAs (Figure 1E, Additional file 1: Figure S1E-F) [9,24]. [file 1471-2164-14-402-S2.pdf]

Figure S3

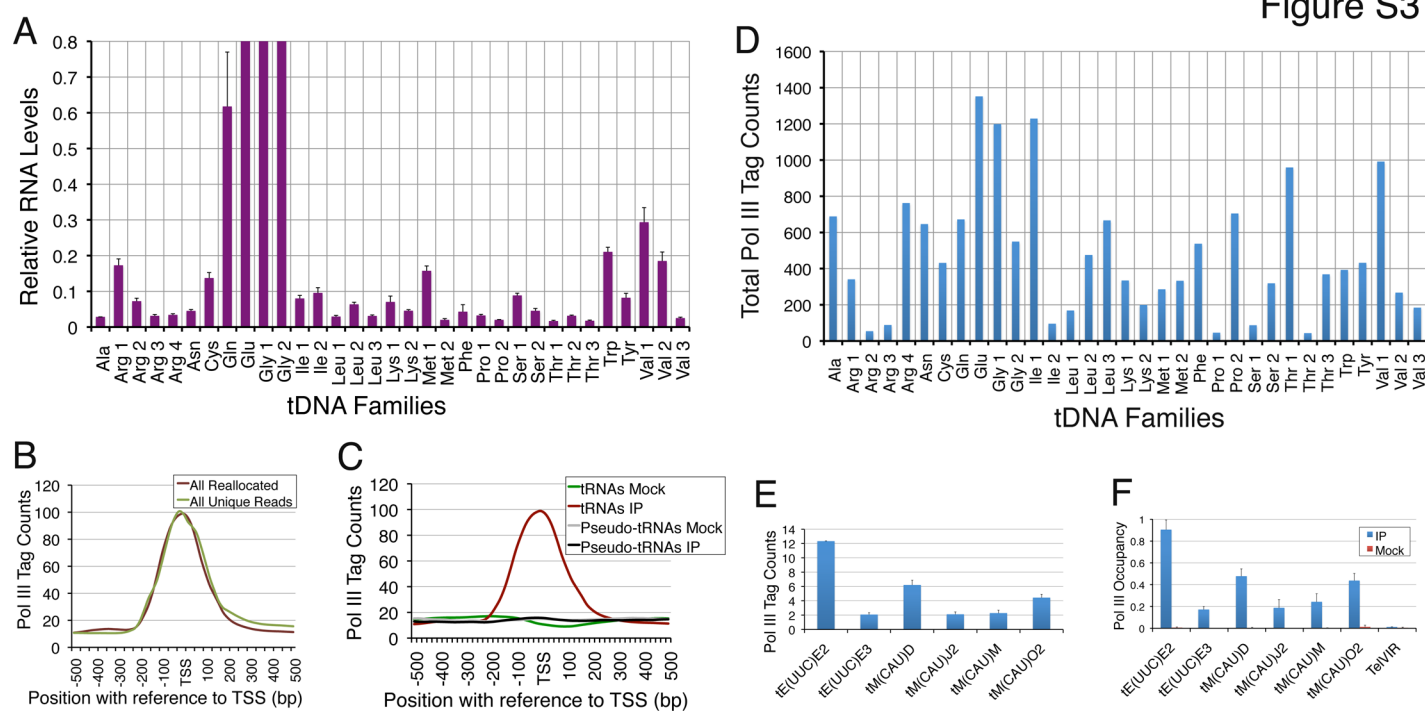

Supplement: Additional file 3: Figure S3 — Pol III and nucleosome occupancy influence transcription. A) RNA levels normalized for copy number of genes by using genomic DNA as calibrator for Real Time PCR quantification of the cDNA for 33 tDNA families, relative to U4 snRNA. Family name descriptions and primer sequences are given in the Additional file 4: Table S3. B) Data analysis using either unique reads only or multi-reads reallocated to the loci, gives similar results. Normalized tag counts from pol III ChIP-seq experiment were calculated in two ways. All unique reads represent tag coverage binned around TSS and averaged for all genes, using only uniquely aligned reads. All reallocated reads represent tag coverage taking unique reads along with non-unique reads that map up to 20 places in genome and reallocating the non- unique reads probabilistically using a previously reported algorithm [30]. Both the plots show high overlap as tRNA genes although repetitive in sequence, are very small in comparison to the ChIP fragment size used in typical ChIP experiments. C) Normalized and averaged tag coverage profiles of all tDNAs and pseudo-tRNA (Additional file 4: Table S2) for RPC128-FLAG IP and mock samples. D) Family wise total pol III tag counts for all the genes that are covered in panel A and Figure 4A. Pol III tag count in a region −100 to +100 bp of every gene was averaged. Then for each primer set, this average pol III count is summed up for all genes that are covered by the primer set. E) Pol III fold-enrichment over mock from ChIP-Seq data for selected genes showing very high to very low pol III levels. F) The data in panel E is validated by ChIP-Real time PCR quantification for pol III on the same genes. Pol III occupancy data for both IP and mock samples are compared with the same on TELVIR region. [file 1471-2164-14-402-S3.pdf]

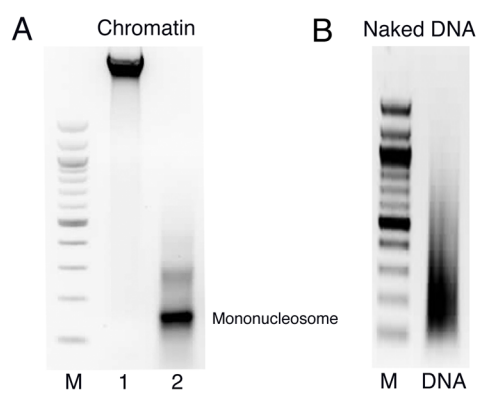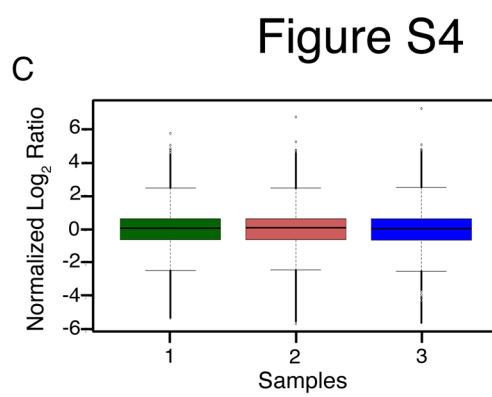

Supplement: Additional file 5: Figure S4 — Quality control of microarray samples and data. (A) Quality check for the mono-nucleosomal DNA preparation. Lane M shows 100 bp DNA ladder used as molecular size marker. Purity of DNA before (lane 1) and after (lane 2) MNase digestion is shown. B) Genomic DNA control for nucleosome mappings after MNase digestion was checked. M shows the 100 bp DNA ladder used as size marker. C) Box plots showing near identical distribution of normalized Log2 ratios among three biological replicates. Pearson correlations between all possible replicate-pairs were 0.97 to 0.99, which confirmed the reproducibility of biological replicates. [file 1471-2164-14-402-S5.pdf]
